# Efficient antibody evasion but reduced ACE2 binding by the emerging SARS-CoV-2 variant B.1.640.2

**DOI:** 10.1038/s41423-022-00870-5

**Published:** 2022-05-17

**Authors:** Prerna Arora, Amy Kempf, Inga Nehlmeier, Luise Graichen, Sebastian Schulz, Anne Cossmann, Alexandra Dopfer-Jablonka, Martin S. Winkler, Hans-Martin Jäck, Georg M. N. Behrens, Stefan Pöhlmann, Markus Hoffmann

**Affiliations:** 1 Infection Biology Unit, German Primate Center, Kellnerweg 4, 37077 Göttingen, Germany; 2 Faculty of Biology and Psychology, Georg-August-University Göttingen, Wilhelmsplatz 1, 37073 Göttingen, Germany; 3 Division of Molecular Immunology, Department of Internal Medicine 3, Friedrich-Alexander University of Erlangen-Nürnberg, Glückstraße 6, 91054 Erlangen, Germany; 4 Department for Rheumatology and Immunology, Hannover Medical School, Carl-Neuberg-Straße 1, 30625 Hannover, Germany; 5 German Centre for Infection Research (DZIF), Partner Site Hannover-Braunschweig, Hannover, Germany; 6 Centre for Individualised Infection Medicine (CiiM), Hannover, Germany; 7 Department of Anesthesiology, University of Göttingen Medical Center, Göttingen, Georg-August University of Göttingen, Robert-Koch-Straße 40, 37075 Göttingen, Germany

**Keywords:** Antibodies, Mechanisms of disease

The SARS-CoV-2 spike (S) protein engages ACE2 for cell entry, and the S protein/ACE2 interface is an important target for neutralizing antibodies. In the course of the COVID-19 pandemic, SARS-CoV-2 variants have emerged that harbor mutations in the S protein, which confer neutralization resistance and allow viral spread in immunologically nonnaive populations. The most prominent example is the highly mutated Omicron variant, which infects convalescent or vaccinated individuals with unprecedented efficiency [1, 2], highlighting the threat that emerging SARS-CoV-2 variants pose to efforts to control the pandemic. Recently, a novel SARS-CoV-2 variant, B.1.640.2, which harbors an unusually high number of mutations in the S protein, was detected in southern France [3]. Here, we analyzed the impact of these mutations on viral cell tropism, ACE2 interactions, and antibody-mediated neutralization.

The B.1.640.2 variant was detected in November 2021 in a vaccinated adult patient who had returned from Cameroon and exhibited mild respiratory symptoms [3]. B.1.640.2 infection was also detected in 12 other individuals living in the same area [3]. The B.1.640.2 S protein analyzed in the present study harbors 13 amino acid substitutions and two deletions (Fig. 1a, b). Three amino acid substitutions and two deletions are located in the N-terminal domain of the S protein (Fig. 1a, b), while six amino acid substitutions are found in the receptor-binding domain (RBD), the portion of the S protein that binds to ACE2 (Fig. 1a, b). While some of these mutations likely alter epitopes for neutralizing antibodies, the Y449N substitution in the RBD is known to impair ACE2 binding [4].

**Fig. 1:**
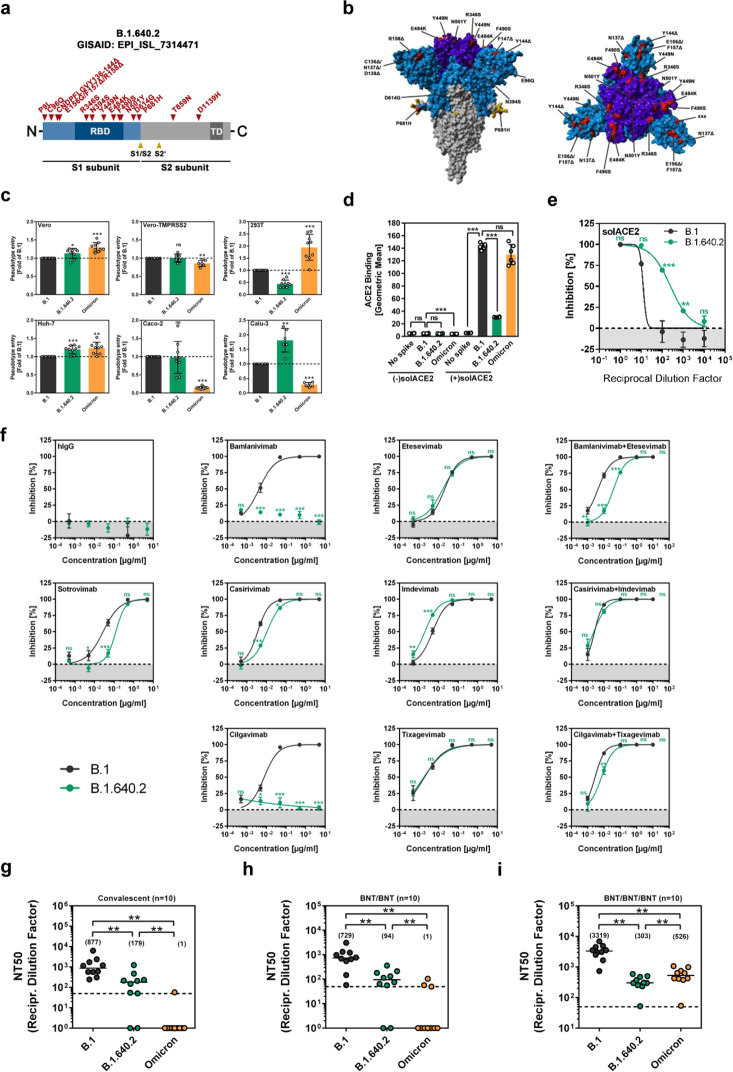
Host cell entry and antibody evasion by SARS-CoV-2 B.1.640.2. **a** Schematic illustration of the SARS-CoV-2 S protein. Mutations found in the S protein of B.1.640.2 (EPI_ISL_7314471) are highlighted in red. RBD receptor-binding domain, TD transmembrane domain. **b** Location of the amino acid changes in the trimeric S protein. **c** Cell lines were inoculated with particles bearing the indicated S proteins. Entry efficiency was analyzed by measuring the activity of virus-encoded luciferase in cell lysates. The average (mean) data ± SEM from six to nine independent experiments are shown; entry driven by the B.1 S protein was set as 1. **d** 293T cells expressing the indicated S protein (or no S protein) were incubated with soluble ACE2 and secondary antibody and analyzed by flow cytometry. Cells incubated with secondary antibodies alone served as controls. The average ± SD geometric mean channel fluorescence from six biological replicates is shown. **e** Particles harboring the indicated S proteins were preincubated with soluble ACE2 before being inoculated into Vero cells. The average (mean) data ± SEM from three biological replicates are shown. **f** Particles bearing the indicated S proteins were incubated with the indicated monoclonal antibody or medium only (control) before being added to Vero cells. Pseudotype entry efficiency was normalized against the respective control (set as 0% inhibition). The average (mean) data ± SEM from three independent experiments are presented. Curves were calculated using a nonlinear regression model (variable slope). **g** Particles bearing the indicated S proteins were incubated with convalescent plasma or medium only (control) before being added to Vero cells. Pseudotype entry was normalized against the respective control (set as 0% inhibition, please see Supplementary Fig. S2a for individual data). Furthermore, the neutralizing titer that reduced pseudotype entry by 50% (NT50) was calculated. The combined data for ten convalescent sera are presented. Black lines and numerical values indicate the median NT50. **h**, **i** The experiment was performed as described in panel **g** except that plasma from vaccinated individuals was analyzed (panel **h**: BNT162b2/BNT162b2, *n* = 10; panel **i**: BNT162b2/BNT162b2/BNT162b2, *n* = 10; please see Supplementary Fig. S2b, c for individual data)

For analysis of the B.1.640.2 S protein, we employed rhabdoviral pseudotypes, which faithfully mimic SARS-CoV-2 entry and antibody-mediated neutralization [5]. We first asked whether particles pseudotyped with B.1.640.2 S protein (B.1.640.2_pp_) can enter cell lines commonly used for SARS-CoV-2 research. The S proteins of variant B.1, which circulated early in the pandemic, and the Omicron variant (sublineage BA.1) served as controls. All particles entered Huh-7 (human liver) and Vero cells (African green monkey kidney) robustly and at comparable rates, and entry efficiency was not increased by the expression of TMPRSS2, an S protein-activating host cell protease (Fig. 1c). Furthermore, B.1.640.2_pp_ entry into 293T cells (human kidney) was reduced, while the entry of Omicron_pp_ was augmented, compared to that of B.1_pp_ (Fig. 1c). Finally, the entry of B.1.640.2_pp_ into Caco-2 (human colon) and Calu-3 (human lung) cells was equal to or more efficient than that measured for B.1_pp_, while the entry of Omicron_pp_ was reduced (Fig. 1c), as previously documented [1]. Thus, the mutations present in the B.1.640.2 S protein can modulate entry into certain cell lines.

We next analyzed the binding of the B.1.640.2 S protein to ACE2, employing S protein-transfected cells and soluble ACE2. The S proteins of B.1 and the Omicron variant bound to ACE2 efficiently, as expected, while binding to B.1.640.2 S protein was markedly reduced (Fig. 1d). The reduced binding of B.1.640.2 S protein to ACE2 correlated with increased inhibition of B.1.640.2_pp_ by soluble ACE2 (Fig. 1e), which is currently being developed as a COVID-19 therapy [6].

Finally, we examined antibody-mediated neutralization. The analysis of monoclonal antibodies that are in development or have already been used for COVID-19 therapy revealed robust inhibition of B.1_pp_ and B.1.640.2_pp_ by most antibodies (Fig. 1f). Bamlanivimab and cilgavimab failed to neutralize B.1.640.2_pp_ (Fig. 1f), in keeping with B.1.640.2 S-specific mutations being located in the respective epitopes (Supplementary Fig. S1). Furthermore, antibodies from convalescent patients neutralized B.1.640.2_pp_ with significantly reduced efficiency compared to B.1_pp_, and neutralization of Omicron_pp_ was further reduced (Fig. 1g, Supplementary Fig. S2a and Supplementary Table S1). Similar results were obtained for sera collected from healthy individuals without prior SARS-CoV-2 infection who had received two doses of the BNT162b2/Comirnaty (BNT/BNT) vaccine (Fig. 1h, Supplementary Fig. S2b and Supplementary Table S2). Neutralization by antibodies in sera collected after the third vaccination with BNT162b2 (BNT/BNT/BNT) was generally more efficient (Fig. 1i and Supplementary Fig. S2c). However, neutralization of B.1.640.2_pp_ was again markedly diminished compared to that of B.1_pp_.

The S protein of variant B.1.640.2 harbors more mutations than its counterparts in the preceding variants of concern, Alpha, Beta, Gamma, and Delta. These mutations had a moderate impact on cell entry but markedly decreased ACE2 binding, potentially due to Y449N [4], and increased inhibition by soluble ACE2. Entry driven by the B.1.640.2 S protein was highly resistant to neutralization by antibodies induced by SARS-CoV-2 infection or BNT162b2 vaccination. This suggests that B.1.640.2 meets an important prerequisite for robust spread in a vaccinated population, namely, antibody evasion, and that reduced ACE2 binding might have impeded such spread.

## Supplementary information

Supplementary material

## References

[1] Hoffmann M, Krüger N, Schulz S, Cossmann A, Rocha C, Kempf A (2022). The Omicron variant is highly resistant against antibody-mediated neutralization: implications for control of the COVID-19 pandemic. Cell.

[2] Vianana R, Moyo S, Amoako DG, Tegally H, Scheepers C, Althaus CL (2022). Rapid epidemic expansion of the SARS-CoV-2 Omicron variant in southern Africa. Nature.

[3] Colson P, Delerce J, Burel E, Dahan J, Jouffret A, Fenollar F (2022). Emergence in southern France of a new SARS-CoV-2 variant harbouring both N501Y and E484K substitutions in the spike protein. Arch Virol.

[4] Tortorici MA, Beltramello M, Lempp FA, Pinto D, Dang HV, Rosen LE (2020). Ultrapotent human antibodies protect against SARS-CoV-2 challenge via multiple mechanisms. Science.

[5] Schäfer A, Muecksch F, Lorenzi JCC, Leist SR, Cipolla M, Bournazos S, et al. Antibody potency, effector function, and combinations in protection and therapy for SARS-CoV-2 infection in vivo. J Exp Med. 2021;218;e20201993. 10.1084/jem.20201993. 10.1084/jem.20201993 PMC7673958 33211088

[6] Monteil V, Kwon H, Prado P, Hagelkrüys A, Wimmer RA, Stahl M (2020). Inhibition of SARS-CoV-2 infections in engineered human tissues using clinical-grade soluble human ACE2. Cell.

