# Supplementary material for: Efficient antibody evasion but reduced ACE2 binding by the emerging SARS-CoV-2 variant B.1.640.2
Source: Cell Mol Immunol. 2022 May 17;19(9):1067–9. doi: 10.1038/s41423-022-00870-5 (PMC9110932; doi:10.1038/s41423-022-00870-5)
Supplement: Supplementary file 1 — Supplementary material [file 41423_2022_870_MOESM1_ESM.pdf]

Supplemental material

Supplemental Material and Methods

Cell culture

293T (human, female, kidney; ACC-635, DSMZ; RRID: CVCL_0063), Vero (African green monkey kidney, female, kidney; CRL-1586, ATCC; RRID: CVCL_0574, kindly provided by Andrea Maisner), Vero cells stably expressing TMPRSS2 (Vero-TMPRSS2) [1] and Huh-7 (human, male, liver; JCRB Cat# JCRB0403; RRID: CVCL 0336, kindly provided by Thomas Pietschmann) were cultured in Dulbecco's modified Eagle medium (DMEM, PAN-Biotech). Additionally, Calu-3 (human, male, lung; HTB-55, ATCC; RRID: CVCL_0609, kindly provided by Stephan Ludwig) and Caco-2 cells (human, male, colon; HTB-37, ATCC, RRID: CVCL_0025) were cultured in minimum essential medium (MEM, GIBCO). All media were supplemented with 10% fetal bovine serum (FBS, Biochrom), 100 U/ml penicillin and 0.1 mg/ml streptomycin (pen/strep) (PAN-Biotech). Vero-TMPRSS2 cells received 1 μg/ml blasticidin (Invivogen). Caco-2 and Calu-3 cells were further supplemented with 1x non-essential amino acid solution (from 100x stock, PAA) and 1 mM sodium pyruvate (PAN-Biotech). All cell lines were incubated at 37 °C in a humidified atmosphere containing 5% CO_2 and were validated by STR-typing, amplification and sequencing of a fragment of the cytochrome c oxidase gene, microscopic examination and/or according to their growth characteristics. Furthermore, all cell lines

were regularly tested for mycoplasma contamination.

Expression plasmids

Expression plasmids pCAGGS-DsRed [1], pCG1-sol-ACE2-Fc [2], pCG1-SARS-CoV-2 B.1 SΔ18 (codon-optimized, C-terminal truncation of 18 amino acid residues, GISAID Accession ID: EPI_ISL_425259) [2] and pCG1-SARS-CoV-2 BA.1 SΔ18 (Omicron variant, codon-optimized, C-terminal truncation of 18 amino acid residues, GISAID Accession ID: EPI_ISL_6640919) [3] have been previously described. The expression plasmids for SARS-CoV-2 B.1.640.2 S (based on GISAID Accession ID: EPI_ISL_7314471), was generated by Gibson assembly using 5 overlapping DNA strings (Thermo Fisher Scientific), linearized (BamHI/XbaI digest) pCG1 plasmid and GeneArt™ Gibson Assembly HiFi Master Mix (Thermo Fisher Scientific). Gibson assembly was performed according to manufacturer's instructions. The pCG1 expression plasmid was kindly provided by Roberto Cattaneo, Mayo Clinic College of Medicine, Rochester, MN, USA. The integrity of all PCR-amplified sequences was verified by sequence analyses performed by a commercial service provider (Microsynth SeqLab).

Sequence analysis and protein models

All S protein sequences and the underlying information (collection date, location) were obtained from the GISAID (global initiative on sharing all influenza data) database (https://www.gisaid.org/). Information on antibody epitopes was obtained from literature [4-]

[9]. Protein models were based on a template in which the SARS-2 S sequence was modelled on PDB: 6XR8 [10] using the SWISS-MODEL online tool (https://swissmodel.expasy.org) and further processed using YASARA (http://www.yasara.org/index.html).

Production of VSV pseudotypes and transduction of target cells

Viral particles pseudotyped with the SARS-CoV-2 S proteins were produced as described previously [11]. In brief, 293T cells were transfected with plasmids encoding S protein or empty plasmid (control) using the calcium phosphate method. At approximately 30 h posttransfection, cells were inoculated with VSV-G-transcomplemented VSV*ΔG(FLuc), a replication-deficient vesicular stomatitis virus (VSV) that lacks the genetic information for its own glycoprotein (VSV-G) and instead codes for two reporter proteins, enhanced green fluorescent protein (eGFP) and firefly luciferase (kindly provided by Gert Zimmer) [12]. After 1 h of incubation, the inoculum was removed and cells were washed with phosphate-buffered saline (PBS). Thereafter, to neutralize residual input virus, all cells received DMEM medium containing anti-VSV-G antibody (culture supernatant from I1-hybridoma cells; ATCC no. CRL-2700) except for cells expressing VSV-G, which received medium without antibody. After an incubation period of 16-18 h, the culture supernatant was harvested, clarified from cellular debris by centrifugation at 4,000 x g for 10 min, aliquoted and stored at -80 °C until further use.

For transduction, target cells seeded in 96-well plates were inoculated with equal volumes of pseudotypes and transduction efficiency was evaluated at 16-18 h post

transduction by measuring luciferase activity in cell lysates. For this, cells were lysed in PBS containing 0.5% Triton X-100 (Carl Roth) for 30 min at room temperature. Subsequently, cell lysates were transferred into white 96-well plates and mixed with luciferase substrate (Beetle-Juice, PJK) before luminescence was measured using a Hidex Sense plate luminometer (Hidex).

Soluble ACE2 binding

The production of soluble ACE2 fused to the Fc portion of human immunoglobulin has been previously described [13]. In order to test binding of soluble ACE2 to the S protein, 293T cells seeded in 6-well plates were transfected with S protein expression plasmids or empty plasmid as negative control. At 24 h posttransfection, the medium was replaced with fresh culture medium. At 48 h posttransfection, the culture medium was aspirated and cells resuspended in PBS and pelleted by centrifugation at 600 x g for 5 min. Subsequently, cells were washed with PBS containing 1 % bovine serum albumin (BSA, PBS-B) and pelleted again. Next, the cell pellets were resuspended in 250 µl PBS-B containing soluble ACE2-Fc (1:25 dilution of 100x concentrated stock) and incubated for 60 min at 4 °C, employing a Rotospin test tube rotator disk (IKA). Thereafter, the cells were pelleted, resuspended in 250 µl PBS-B containing anti-human AlexaFlour-488-conjugated antibody (1:200; Thermo Fisher Scientific) and incubated again for 60 min at 4 °C. After a final wash step with PBS-B, the cells were fixed with 4 % paraformaldehyde solution for 30 min at RT, washed, resuspended in 150 µl PBS-B and analyzed by flow cytometry, using an LSR II flow

cytometer and Flowing software version 2.5.1 (https://bioscience.fi/services/cell-imaging/flowing-software/).

Inhibition of S protein-driven cell entry by soluble ACE2

S protein bearing particles were pre-incubated for 30 min at 37 °C with different dilutions of soluble ACE2 (undiluted [1:100 dilution of 100x concentrated stock], 1:10, 1:100, 1:1,000, 1:10,000). After incubation, the mixtures were added to Vero cells. Particles exposed to medium without soluble ACE2 served as control. Transduction efficiency was determined at 16-18 h postinoculation by determining luciferase activities in cell lysates, as described above.

VSV pseudotype-based neutralization assay

Collection of convalescent plasma and vaccinee serum samples has been described before [2,14,15]. Convalescent plasma was obtained from patients treated at the University Medicine Göttingen (UMG) and vaccinee serum was obtained at the Hannover Medical School (Medizinische Hochschule Hannover, MHH). Sample collection was approved by the research ethics committee of the UMG (SeptImmun Study and PneumoSept Study) and the Institutional Review Board of MHH (8973_BO_K_2020). All serum and plasma samples were heat-inactivated at 56 °C for 30 min. Neutralization assays were conducted as described [14,15]. In brief, S protein bearing particles were pre-incubated for 30 min at 37 °C with different concentrations (5, 0.5, 0.05, 0.005, 0.0005 µg/ml; of note, for antibody

cocktails each antibody was used at half of the indicated concentration to keep total antibody concentration constant) of SARS-CoV-2-specific monoclonal antibody (Casirivimab, Imdevimab, Bamlanivimab, Etesevimab, Sotrovimab, Cilgavimab, Tixagevimab) and combinations (Casirivimab + Imdevimab, Bamlanivimab + Etesevimab, Cilgavimab + Tixagevimab) or an unrelated control antibody (hIgG). Particles incubated with medium alone served as control (= 0% inhibition). Alternatively, pseudotyped particles were pre-incubated with different dilutions of convalescent plasma (1:50, 1:200, 1:800, 1:3,200 and 1:12,800) or serum from individuals vaccinated twice or thrice with the BNT162b2/Comirnaty (BNT) vaccine (1:50, 1:200, 1:800, 1:3,200 and 1:12,800). Following incubation, mixtures were inoculated onto Vero cells with particles incubated with medium alone serving as control (= 0% inhibition). Transduction efficiency was determined at 16-18 h postinoculation as described above.

Statistical analysis

Data were analyzed using Microsoft Excel (as part of the Microsoft Office Professional Plus, version 2016, Microsoft Corporation) and GraphPad Prism 6 version 6.07 (GraphPad Software). Statistical significance was assessed by two-tailed Students t-test with Welch's correction (panel c) or by two-way analysis of variance with Sidak's post hoc tests (panel d-f) or by Wilcoxon matched-pairs signed rank test (panel g-i). Only p-values 0.05 or lower were considered statistically significant ($p > 0.05$, not significant [ns]; $p \leq 0.05$, *; $p \leq 0.01$, **; $p \leq 0.001$, ***). Plasma/Serum dilutions that lead to a 50 % reduction of transduction

efficiency (neutralizing titer 50, NT50), were calculated by a non-linear regression model with variable slope.

REFERENCES

1 Hoffmann, M. *et al.* SARS-CoV-2 Cell Entry Depends on ACE2 and TMPRSS2 and Is Blocked by a Clinically Proven Protease Inhibitor. *Cell* **181**, 271-280 e278, doi:10.1016/j.cell.2020.02.052 (2020).

2 Hoffmann, M. *et al.* SARS-CoV-2 variants B.1.351 and P.1 escape from neutralizing antibodies. *Cell* **184**, 2384-2393 e2312, doi:10.1016/j.cell.2021.03.036 (2021).

3 Hoffmann, M. *et al.* The Omicron variant is highly resistant against antibody-mediated neutralization: Implications for control of the COVID-19 pandemic. *Cell* **185**, 447-456 e411, doi:10.1016/j.cell.2021.12.032 (2022).

4 Dong, J. *et al.* Genetic and structural basis for SARS-CoV-2 variant neutralization by a two-antibody cocktail. *Nat Microbiol* **6**, 1233-1244, doi:10.1038/s41564-021-00972-2 (2021).

5 Hansen, J. *et al.* Studies in humanized mice and convalescent humans yield a SARS-CoV-2 antibody cocktail. *Science* **369**, 1010-1014, doi:10.1126/science.abd0827 (2020).

6 Jones, B. E. *et al.* The neutralizing antibody, LY-CoV555, protects against SARS-CoV-2 infection in nonhuman primates. *Sci Transl Med* **13**, doi:10.1126/scitranslmed.abf1906 (2021).

7 Kim, C. *et al.* A therapeutic neutralizing antibody targeting receptor binding domain of SARS-CoV-2 spike protein. *Nat Commun* **12**, 288, doi:10.1038/s41467-020-20602-5 (2021).

8 Pinto, D. *et al.* Cross-neutralization of SARS-CoV-2 by a human monoclonal SARS-CoV antibody. *Nature* **583**, 290-295, doi:10.1038/s41586-020-2349-y (2020).

9 Shi, R. *et al.* A human neutralizing antibody targets the receptor-binding site of SARS-CoV-2. *Nature* **584**, 120-124, doi:10.1038/s41586-020-2381-y (2020).

10 Cai, Y. *et al.* Distinct conformational states of SARS-CoV-2 spike protein. *Science* **369**, 1586-1592, doi:10.1126/science.abd4251 (2020).

11 Kleine-Weber, H. *et al.* Mutations in the Spike Protein of Middle East Respiratory Syndrome Coronavirus Transmitted in Korea Increase Resistance to Antibody-Mediated Neutralization. *Journal of virology* **93**, doi:10.1128/JVI.01381-18 (2019).

12 Berger Rentsch, M. & Zimmer, G. A vesicular stomatitis virus replicon-based bioassay for the rapid and sensitive determination of multi-species type I interferon. *PloS one* **6**, e25858, doi:10.1371/journal.pone.0025858 (2011).

13 Hoffmann, M., Kleine-Weber, H. & Pohlmann, S. A Multibasic Cleavage Site in the Spike Protein of SARS-CoV-2 Is Essential for Infection of Human Lung Cells. *Mol Cell* **78**, 779-784 e775, doi:10.1016/j.molcel.2020.04.022 (2020).

14 Arora, P. *et al.* B.1.617.2 enters and fuses lung cells with increased efficiency and evades antibodies induced by infection and vaccination. *Cell reports* **37**, 109825, doi:10.1016/j.celrep.2021.109825 (2021).

15 Arora, P. *et al.* The spike protein of SARS-CoV-2 variant A.30 is heavily mutated and evades vaccine-induced antibodies with high efficiency. *Cell Mol Immunol*, doi:10.1038/s41423-021-00779-5 (2021).

16 WHO Working Group on the Clinical Characterisation Management of Covid-infection. A minimal common outcome measure set for COVID-19 clinical research. *Lancet Infect Dis* **20**, e192-e197, doi:10.1016/S1473-3099(20)30483-7 (2020).

Supplemental tables

Supplemental table 1. COVID-19 patients

Identifier	Infecting strain	Gender	Age group (years)	Period	Symptoms
**SI15**	Unknown	Male	65-74	1st wave	Severe*
**SI18**	Unknown	Female	65-74	1st wave	Severe*
**SI20**	Unknown	Male	55-64	1st wave	Severe*
**SI22**	Unknown	Female	25-34	1st wave	Severe*
**SI23**	Unknown	Female	65-74	2nd wave	Severe*
**SI24**	Unknown	Male	55-64	2nd wave	Severe*
**SI27**	Unknown	Male	45-54	2nd wave	Severe*
**SI33**	Unknown	Male	75-84	2nd wave	Severe*
**SI51**	Unknown	Male	65-74	2nd wave	Severe*
**SI56**	Unknown	Male	65-74	2nd wave	Severe*

*: According to the guidelines of the WHO Working Group on the Clinical Characterisation and Management of COVID-19 infection [16].

186 **Supplemental table 2. Vaccinees**

Identifier	Vaccination	Gender	Age group (years)	Documented SARS-CoV-2 infection? (yes/no)	Time between 1st and 2nd vaccination (days)	Time between 2nd and 3rd vaccination (days)	Time since last vaccination (days)
**4803**	BNT/BNT	Female	35-44	no*	17	n/a	13
**4811**	BNT/BNT	Female	25-34	no*	28	n/a	15
**4828**	BNT/BNT	Female	18-24	no*	21	n/a	27
**4848**	BNT/BNT	Male	25-34	no*	20	n/a	25
**4863**	BNT/BNT	Female	55-64	no*	21	n/a	25
**4866**	BNT/BNT	Female	55-64	no*	25	n/a	27
**4874**	BNT/BNT	Female	25-34	no*	27	n/a	27
**4877**	BNT/BNT	Female	25-34	no*	29	n/a	27
**4883**	BNT/BNT	Female	35-44	no*	26	n/a	34
**4900**	BNT/BNT	Female	45-54	no*	22	n/a	28
**7423**	BNT/BNT/BNT	Male	25-34	no*	23	244	19
**7426**	BNT/BNT/BNT	Male	55-64	no*	21	237	19
**7532**	BNT/BNT/BNT	Female	45-54	no*	22	255	19
**7533**	BNT/BNT/BNT	Male	35-44	no*	21	257	17
**7539**	BNT/BNT/BNT	Female	25-34	no*	21	262	14
**7562**	BNT/BNT/BNT	Male	25-34	no*	19	258	24
**7580**	BNT/BNT/BNT	Female	45-54	no*	23	264	20
**7585**	BNT/BNT/BNT	Female	45-54	no*	22	262	24
**7595**	BNT/BNT/BNT	Female	55-64	no*	26	261	26
**7773**	BNT/BNT/BNT	Female	35-44	no*	17	264	21

187 *: Negative SARS-CoV-2 infection status of vaccinees was confirmed by negative antigen tests for
188 SARS-CoV-2 spike (before vaccination) and nucleoprotein (after last vaccination).
189 Abbreviations: BNT, BNT162b2; n/a, not applicable

Supplemental figures

Supplemental figure 1

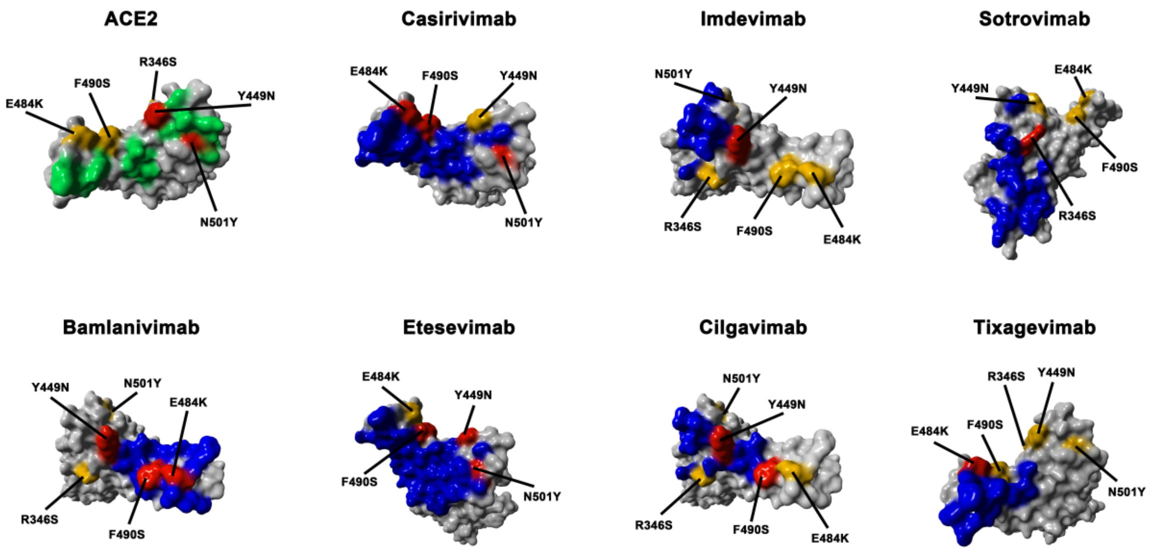

Supplemental figure 1. Epitopes of neutralizing monoclonal antibodies and location of B.1.640.2 S protein specific mutations. Locations of B.1.640.2-specific mutations in the context of the RBD epitopes targeted by ACE2, casirivimab, imdevimab, sotrovimab, bamlanivimab, etesevimab, cilgavimab and tixagevimab. (RBD, gray; epitope targeted by the antibody, blue; B.1.640.2-specific mutations within the epitope, red; B.1.640.2-specific mutations outside the epitope, orange) and amino acid residues that directly interact with ACE2 (green).

Supplemental figure 2

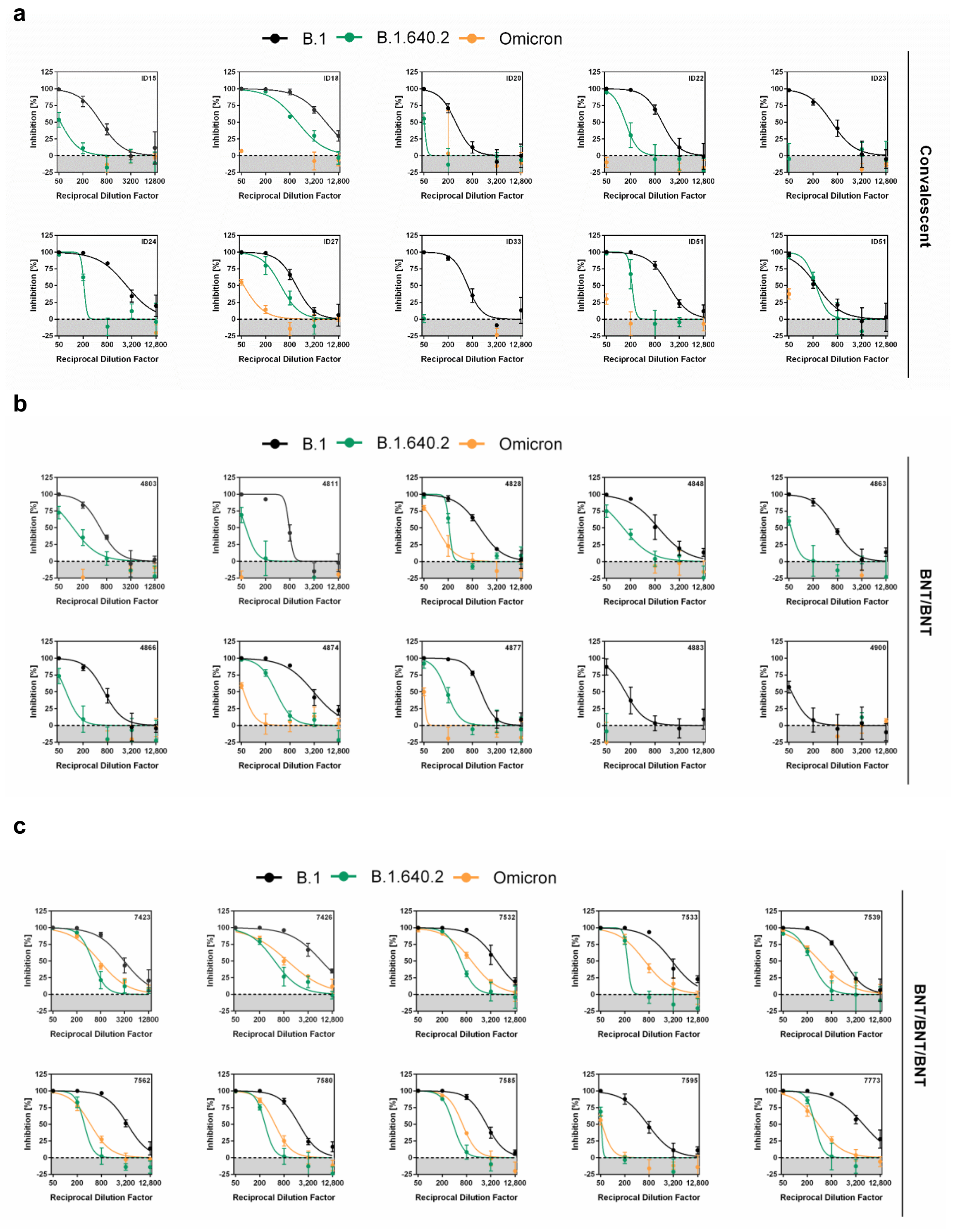

Supplemental figure 2. Individual neutralization data for each convalescent plasma (a), as well as BNT/BNT (b) and BNT/BNT/BNT (c) vaccinee serum. Data represent mean of four technical replicates with standard deviation (error bars). Data were normalized against control samples without serum (0% inhibition). Curves were calculated using a non-linear regression model with variable slope.
